# Entrapment Bias of Arthropods in Miocene Amber Revealed by Trapping Experiments in a Tropical Forest in Chiapas, Mexico

**DOI:** 10.1371/journal.pone.0118820

**Published:** 2015-03-18

**Authors:** Mónica M. Solórzano Kraemer, Atahualpa S. Kraemer, Frauke Stebner, Daniel J. Bickel, Jes Rust

**Affiliations:** 1 Steinmann-Institut für Geologie, Mineralogie und Paläontologie, Rheinische Friedrich–Wilhelms-Universität Bonn, Bonn, Germany; 2 Departamento de Física, Facultad de Ciencias, Universidad Nacional Autónoma de México (UNAM), Ciudad Universitaria, Distrito Federal Mexico, Mexico; 3 Senckenberg Forschungsinstitut und Naturmuseum, Frankfurt am Main, Germany; 4 Australian Museum, Sydney NSW 2010, Australia; University of Tours, FRANCE

## Abstract

All entomological traps have a capturing bias, and amber, viewed as a trap, is no exception. Thus the fauna trapped in amber does not represent the total existing fauna of the former amber forest, rather the fauna living in and around the resin producing tree. In this paper we compare arthropods from a forest very similar to the reconstruction of the Miocene Mexican amber forest, and determine the bias of different trapping methods, including amber. We also show, using cluster analyses, measurements of the trapped arthropods, and guild distribution, that the amber trap is a complex entomological trap not comparable with a single artificial trap. At the order level, the most similar trap to amber is the sticky trap. However, in the case of Diptera, at the family level, the Malaise trap is also very similar to amber. Amber captured a higher diversity of arthropods than each of the artificial traps, based on our study of Mexican amber from the Middle Miocene, a time of climate optimum, where temperature and humidity were probably higher than in modern Central America. We conclude that the size bias is qualitatively independent of the kind of trap for non–extreme values. We suggest that frequent specimens in amber were not necessarily the most frequent arthropods in the former amber forest. Selected taxa with higher numbers of specimens appear in amber because of their ecology and behavior, usually closely related with a tree–inhabiting life. Finally, changes of diversity from the Middle Miocene to Recent time in Central and South America can be analyzed by comparing the rich amber faunas from Mexico and the Dominican Republic with the fauna trapped using sticky and Malaise traps in Central America.

## Introduction

The study of the evolution of ecosystems over geological time is one of the most intricate topics in paleontological research, especially in terrestrial tropical areas with a very high organismal diversity and usually a very poor fossil record. Among the most important and most challenging aspects of investigations into the evolution of paleodiversity in tropical realms is the impact of taphonomic processes on the fossilisation in former ecosystems. The knowledge of what is being conserved or lost under specific conditions of fossilization is essential for interpreting the former ecosystem (e.g. [1]). Arthropods play a dominant role in extant tropical forests, but the history of their present diversity as documented in the fossil record is limited due to specialized fossilisation conditions found in specific deposits like lacustrine or marine sediments and especially amber deposits (e.g. [2]). Fossils in amber are well known for their exceptionally detailed preservation and species richness. They are among the most important resources for the reconstruction of former terrestrial environments, especially in tropical and subtropical regions like those represented by the Dominican and Mexican ambers (e.g. [2–9]; among others). Two of the most comprehensive analyses and complete reconstructions of a former amber forest were carried out by Larsson [10] on Eocene Baltic amber, and by Poinar & Poinar [7] on Middle Miocene Dominican amber. However, even the well investigated Baltic amber represents only a fraction of the biota that lived in the former amber forest.

Amber contains organisms from different habitats such as tree bark, soil and litter, freshwater, and even marine littoral realms, due to differences in the process of maturation of the resin [11] and due to rare entrapment events over time.

The entrapment of arthropods from such different local environments in resin is a highly selective process, depending on factors like the resin producing tree, the size and behavior of arthropods, seasonality and regional climatic gradients, local and regional environment, resin composition, and insect dehydration [1]. However, few taphonomic analyses of amber faunas have been done so far (e.g. [12]). The first major study was that of Brues [13], who compared the fauna of Baltic amber with arthropods trapped from a forest near Petersham, Massachusetts with flypaper, a paper coated with a sticky mixture of castor oil, resins, and wax. Brues [13] believed that the flypaper had the same sampling properties as resin and furthermore he assumed that the ecological conditions of the former Baltic amber forest would be similar to the Petersham forest. Consequently, he concluded that the insect fauna had changed considerably over time. However, his basic assumptions were rejected later by Henwood [14]. She compared extant faunas from different Neotropical rain forests collected with different kinds of traps in Brazil and Mexico with the amber fauna from Miocene Dominican amber. Henwood [14] concluded that the fauna trapped with emergence traps was most similar to the Dominican amber fauna and that amber reflects ground–subterranean level resin production. These conclusions were rejected by Penney [15], who demonstrated that at least in recent Neotropical rainforests the spider fauna from Dominican amber is most closely related to extant tree–inhabiting species away from the ground layer. In one of the most recent works about insects in contemporary resins, Zherikhin et al. [16] compared the fauna of Recent resins collected from different Pinaceae in many localities across northern Eurasia with Eocene Baltic, Rovno, Bitterfeld and Miocene Dominican ambers. They found a general trend towards a decrease in the relative abundance of living arboreal springtails and nematoceran flies and an increase in that of true bugs, beetles, lepidopterans, and hymenopterans in comparison with the amber. Penney and Langan [17] compared for the first time the size of spiders from Dominican and Baltic ambers showing that overall, the body size of web–spinning spiders in Baltic amber was greater than in Dominican amber, while for active hunting spiders the size was similar. They concluded that the behavior of the spiders and morphology of the resin producing tree are more important factors for the entrapment than the size of the organism. Generally it is considered that small–sized arthropods are more likely to get trapped in resin; however this has never been studied in detail for all arthropods in amber.

**Fig 1:**
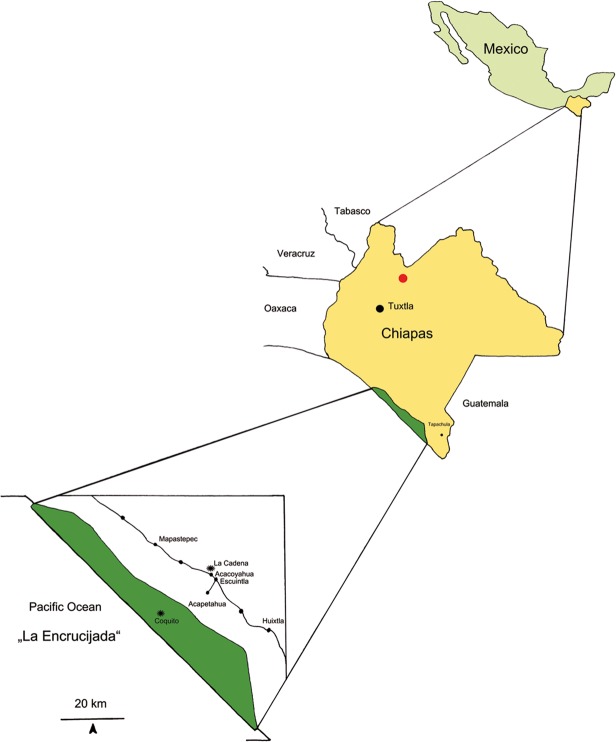
Location of collecting sites “Coquitos” and “La Cadena” in Chiapas, Mexico and in red location of the most important amber mines, Simojovel, Chiapas.

Arthropods found in Tertiary ambers are generally closely related to living representatives, but today, resinous forests similar to the Tertiary amber forests (e.g., Baltic amber, Sicilian amber) are non–existent, or they have been altered by strong anthropogenic impact (Dominican amber). One exception is Mexican amber. The extant forest of the Pacific coast of Southern Mexico, in the state of Chiapas, in the biosphere reserve “La Encrucijada” (Fig. 1), shows great similarity of floral composition with the former amber forest, characterized as a tropical lowland area close to mangroves [8, 18]. In this area, almost all of the living plants were also recognized by Graham [19], Martínez–Hernández [20] and Langenheim [18] from the palynological record of the amber bearing sediments [8]. This includes the genus *Hymenaea*, which is the original resin producing tree for the Mexican and Dominican ambers and copal from many regions in South America, Madagascar and East Africa. The diversity of the extant flora of Chiapas has been compiled by Breedlove [21]; additionally, the flora and fauna from the biosphere reserve were summarized by Carabias Lillo et al. [22]. The conspicuous similarity of the floral composition of these extant forests to the former forests of Mexican amber provided the opportunity to compare the living fauna with the fossil amber fauna, to address analyses of the taphonomical biases and filters during the fossilization process.

Mexican and Dominican ambers belong to the Middle Miocene [8, 23–25], a period of warm and optimal climate conditions for many organisms. Mexican amber is one of the most important amber deposits of the world, and together with Dominican amber, it provides detailed information about the evolution of biodiversity and the development of terrestrial ecosystems in the Central American and Caribbean area. However, for a comprehensive reconstruction of the ancient amber forest it is necessary to obtain not only systematic, palaeoecological and biogeographical information from the amber and its inclusions, but also a better understanding of the taphonomic processes that transfer a diverse living community into a fragmentary fossil taphocoenosis.

To study those processes more than 50,000 recent arthropods have been collected from seven different types of traps placed in two collection sites in Chiapas, Mexico and compared to almost 3,000 Mexican amber fossil arthropod specimens from various institutions. Besides taphonomic studies, the present work is also an analysis of the arthropod diversity of the biosphere reserve forest as an intrinsic result of the collecting work. The distribution of the collected fauna was studied in two different collection areas in order to characterize special habitats and compare them with the fauna from the former Mexican amber forest. Since the habitat characterization depends also on the season, the fieldwork was carried out during the two principal seasons, the rainy season and the dry season; the conditions differed mainly in temperature, humidity, duration of sunlight, number of predators, and life cycles of some arthropods.

The present work highlights the difficulty of finding a trap resembling amber–forming resin and its function as an entomological trap, and the degree to which the amber fauna is representative of the total potential faunal diversity from a former amber forest. Based on the similarity of the former Miocene Mexican amber forest with the lowland forest in the south of Mexico we test different hypotheses on the bias of amber faunas more rigorously comparing the following variables: 1) collecting sites, how different or similar are both collecting places in comparison with the amber fauna; 2) dry or rainy season, how important is seasonality with respect to biases of different traps; 3) peculiarities of different types of traps, 4) size distribution and guilds of trapped arthropods compared with amber. Although, the present study had the intention to compare inclusions in amber with inclusion in fresh resin too. Although, this was not possible because of the small amount of resin production in the tested trees.

## Material and Methods

### Amber

The 2,824 Mexican amber inclusions used in this research for comparison with the entrapped arthropods belong to four collections: (1) Staatliches Museum für Naturkunde, (SMNS), Stuttgart, Germany; (2) University of California, Museum of Paleontology (UCMP), Berkeley, California, USA; (3) Museo de Paleontología (IHNE), Chiapas, Mexico; and (4) Naturmuseum Senckenberg (SMF), Frankfurt, Germany. The largest part of the worldwide available fossil material from Mexican amber has already been intensively studied by Solórzano Kraemer [8]. However, two kilograms of new material of Totolapa amber (containing 107 specimens), and 37 pieces of Palenque amber (containing 226 specimens) from Chiapas, Mexico have been added from a new small collection acquired by the Naturmuseum Senckenberg, Germany.

For the present work, we take all available data on Mexican amber from different museums, where the collections were taken without emphasis on special taxa or any other previous selection (SMNS collection and Totolapa and Palenque amber). Data on Dominican amber spiders have been taken from Penney [15], because the data from the Mexican amber spiders were not sufficient to gain a statistically significant result. Dolichopodidae from Dominican amber were surveyed by DJB from the American Museum of National History (AMNH), New York. Data on Dominican amber Psychodidae are from Grund [26] and EDNA.

For the analysis of the amber collection the possible bias factor of anthropogenic selection of special pieces of amber (e.g., with respect to size and quality of inclusions) must be considered. Pike [28] has discussed the impact of collecting bias in detail. For the present study the method of amber sampling could not be chosen, since it is not possible to collect an adequate amount of amber with inclusions in the field. However, the anthropogenic bias in the collection of amber can be controlled to some degree by using unbiased collections for comparison, which avoids both intentional and unintentional selection of material (e.g. [27, 29]).

### Recent arthropods

The entrapment experiments on recent arthropods were carried out in two areas. The first one is localized within the Biosphere Reserve “La Encrucijada” (Fig. 1) at the southern coast of Chiapas, Mexico, referred to by the residents as “Coquitos”. This region is located very close to the coast, between mangroves in a zone of conservation (ZC). This part of the reserve is characterized by evergreen seasonal forest. In this area, arthropods were collected on and around *Bursera* trees, chosen for the present study because of the absence of *Hymenaea* in this area and because it is also a resin producing tree. The second collection area is located on the edges of the reserve and is called “La Cadena”. In this area, arthropods were collected on and around *Hymenaea* trees close to Acacoyahua 15° 21’ N, 92° 41’ W. Acacoyahua is located 70m above sea level; La Cadena is not a protected area and is characterized by evergreen forest but also by plantations of cocoa and some other agricultural activities. However, not many human communities exist in this zone and a large area of forest remains intact. The distance between both collection zones is approximately 25 km. Mean annual temperature in Coquitos is 28°C and in La Cadena 27°C. The annual precipitation in Coquitos is 2150 mm, in La Cadena 3600 mm. The rainy season is well delimited between May and October.

### Collection methods

**Fig 2:**
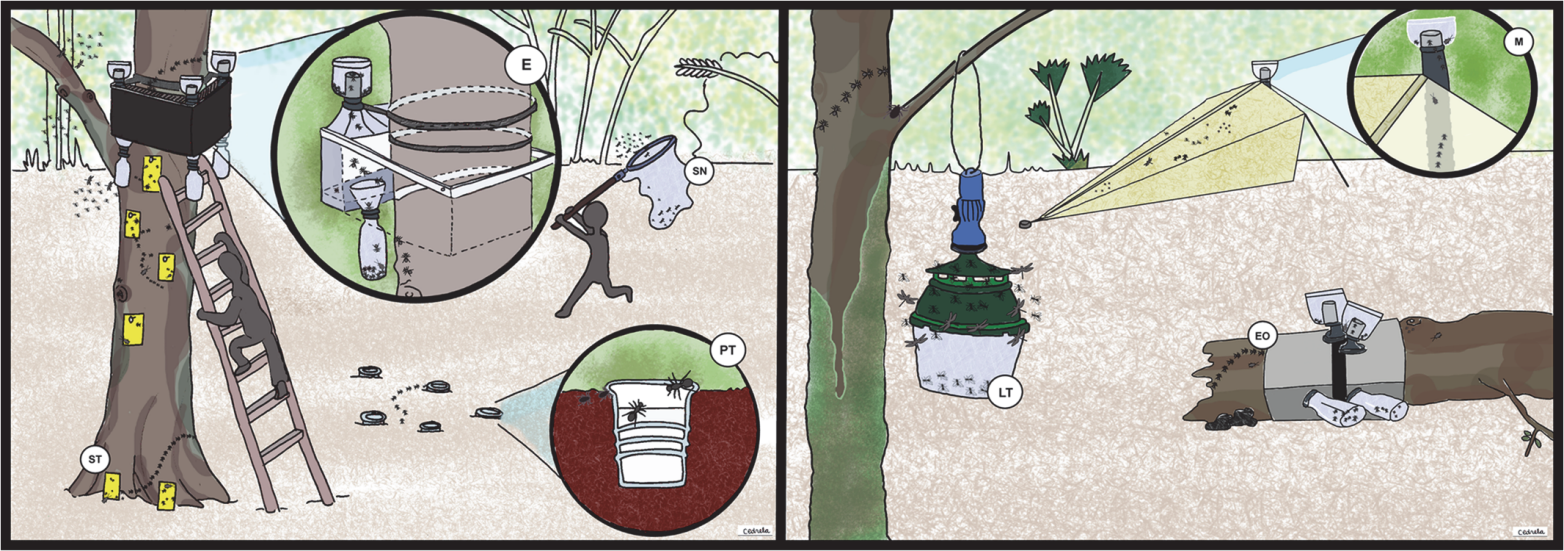
Graphic representation of the used traps. ST Sticky trap, EO Eclector trap open, E Eclector trap, M Malaise trap, PT Pitfall trap, LT Light trap, SN Sweep netting.

The following seven collection methods, represented in the Fig. 2, have been used in both collection sites:

1. Arboreal photo–eclector produced by EcoTech (hereafter named “eclector”): The eclector traps were chosen since they are suitable for catching a wide range of arthropods on tree trunks (e.g., wood–inhabiting species and species moving around on bark, branches, etc.). The eclector operates with a high–grade square framework of steel that is fastened over the holding device to the trunk. Animals that fall within the eclectors of the trunk or the side panels of the trap system can likewise be seized. The eclector traps used for standing and fallen trunks have a diameter ranging from 0.2 to 0.8m.
2. Sticky trap: The 7.5 x 20 cm yellow and transparent sticky traps, odourless and with an insecticide–free sticky mixture are especially suitable for catching the fauna around the trees. They can be acquired from agronomic product stores. Sticky traps were placed homogeneously from the base to three meters high in each tree. Arthropods from the sticky traps were first dissolved in gasoline and then transferred to ethanol. Because yellow and transparent were not statistically different, both are hereafter named “sticky trap”.
3. Pitfall trap: For pitfall traps we used small plastic cups, which are easy to find everywhere. They were buried in the soil around the tested trees. They are especially suitable for catching ground–dwelling arthropods, particularly springtails and ground beetles but also mites and ants.
4. Open eclectors produced by EcoTech: The open eclector is a kind of eclector made for trees that are fallen on the ground. Like the photo–eclector, an open eclector catches a wide range of arthropods, such as those living in dead tree trunks.
5. Malaise trap: Malaise traps were acquired from Bioform. A Malaise trap is a large, tent–like structure trap that was chosen because it is especially suitable for catching a wide range of principally flying insects like Hymenoptera and Diptera. Malaise traps were located very close to the trees and about 20 m from water bodies.
6. Light trap: The light traps were constructed with a hanging bucket trap acquired from Bioform and LSD lamps with batteries for two days. They were cleaned and the lamp was changed every two days. Light traps were chosen because they attract nocturnal active flying and terrestrial arthropods. In combination with hanging buckets it was possible to have small light traps in all the investigated trees.
7. Sweep netting: Traditional sweep netting samples were used for collecting arthropods around the trees up to a distance of about 5 m or more, in order to consider the fauna of the associated vegetation. Collection by this method was possible only during the day and during the time we visited the collection places.

One eclector and one Malaise trap were installed, along with four light traps, ten sticky traps collocated on the trunk and branches, and four pitfall traps for each tree. The killing agent for eclectors, Malaise, Light trap, and pitfall trap was alcohol. The eclectors were placed on the principal trunk at about 4 m height. The traps were installed on each tree for two weeks and specimens were collected every two days. Sticky traps were installed for one week. Three *Hymenaea courbaril* trees in La Cadena and three *Bursera simaruba* trees in Coquitos have been chosen for performing the trapping experiments. The *Bursera* trees were growing very close to the mangrove swamps and the *Hymenaea* trees grow about 25 km away, near the border of the Biosphere Reserve and close to a river. Finally, arthropods were also collected with “open eclectors” from a fallen trunk found in each collected area. The trees were between 20 and 30 m apart from each other. Three collection trips were made: one during the dry season (April 2011) and two during the rainy season (June–July 2010, May–June 2012), covering the two most important seasons of the region. For the recent collections the same conditions were maintained for all traps as far as it was possible. The arthropods were stored in glass–screw–top vials in 70% alcohol. All the specimens were sorted at the order level; for Diptera and Arachnida also families, for the family Formicidae (Hymenoptera) subfamilies and for the families Dolichopodidae (Diptera) and Psychodidae (Diptera) even genera have been identified (see Tables A–F in S1 File). The body, from the head to the end of abdomen, of Recent and fossil specimens was measured. For measurement a progressive classification was used: 0–1 mm, 1–2 mm, 2–3 mm, 3–4…. to 30 mm, which is the largest size of an arthropod found in the analyzed collection. For guild classification we used the method established by Moran and Southwood [30]. The National Institute of Ecology (SEMARNAT) in Mexico, gave permission for the collection of living arthropods for the work presented here. All arthropods will finally be housed in the Biology Department at the National University of Mexico (UNAM), Mexico City, curated by Prof. Juan J. Morrone.

### Comparisons

**Fig 3:**
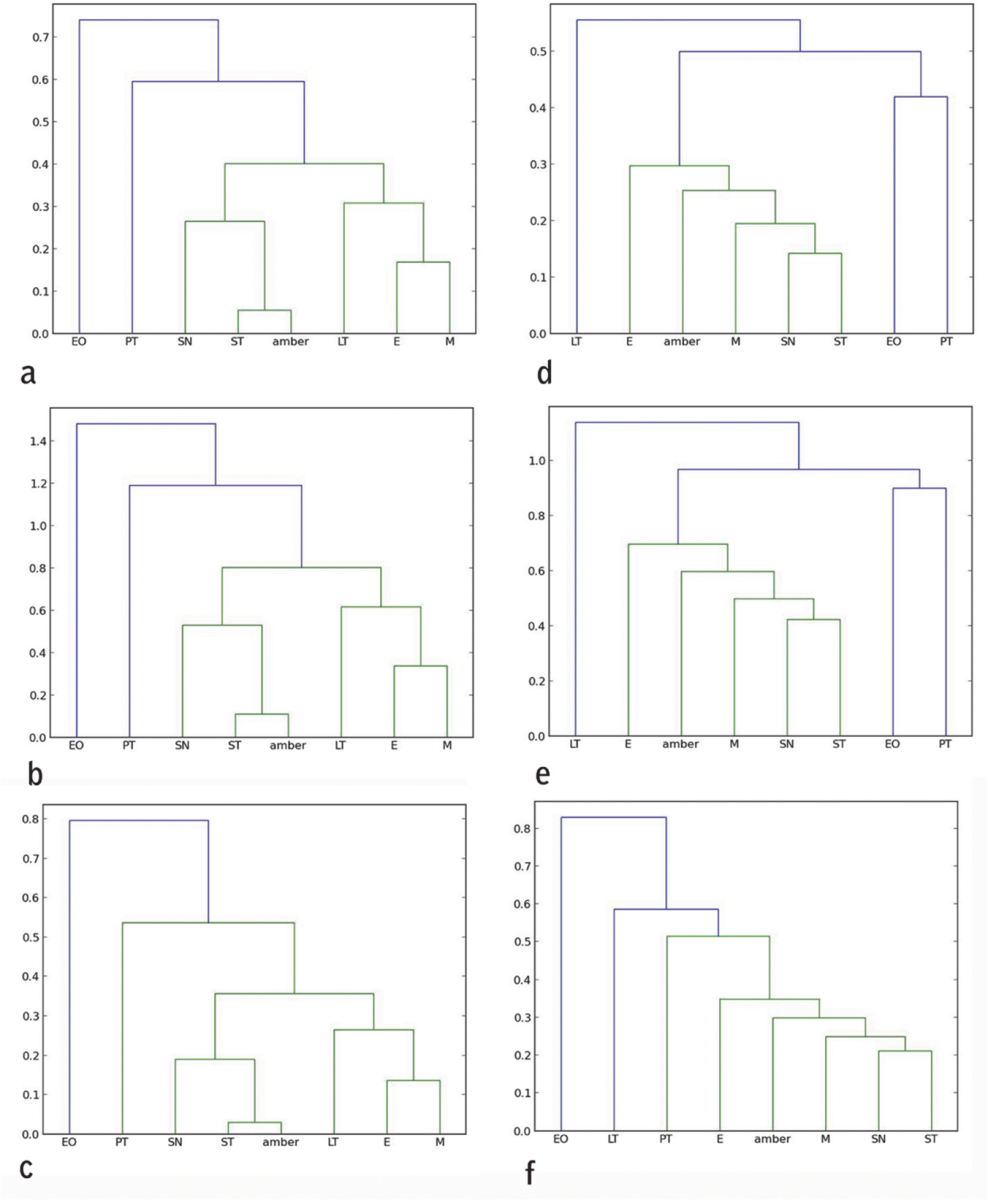
Cluster analysis to compare all entomological traps with the amber. a) Manhattan distance for all orders collected in Coquitos, b) Bray–Curtis for all orders collected in Coquitos, c) Euclidean distance for all orders collected in Coquitos d) Manhattan distance for all orders collected in La Cadena, e) Bray–Curtis for all orders collected in La Cadena, f) Euclidian distance for all orders collected in La Cadena. ST Sticky trap, EO Eclector trap open, E Eclector trap, M Malaise trap, PT Pitfall trap, LT Light trap, SN Sweep netting.

Cluster analysis. Amber data and living insect data were included in a cluster analysis to compare all entomological traps with the amber trap. Following Henwood [14], the cluster analysis distances between samples were calculated using the Manhattan distance and the method used was the UPGMC, which is the most suitable method to compare two populations [14]. However, to compare our results with Penny [15] we used also the Bray–Curtis index, which is a modified Manhattan measurement. Bray–Curtis index differs only by a constant, in case of comparing percentages the constant is two and the Euclidean distance, which is usually not taken into account because of the lack of data; this method is more sensitive to the dissimilarity. In addition, Euclidian measure is a direct measure of the dissimilarity, such that this analysis can be used to qualify the data by comparing the three methods. Fig. 3 shows the clustering analysis for the three different methods for the two chosen ecological regions in Chiapas.

To estimate the degrees for diversity of the different traps, we used rarefaction analysis (see [31]). This method consists of generating a theoretical curve that estimates the number of taxa found. For this estimation, the number of specimens within the collection is taken into account. With this theoretical curve it is possible to compare the quantity of taxa for all collection methods, even if the number of collected specimens is different.

## Results and Discussion

**Fig 4:**
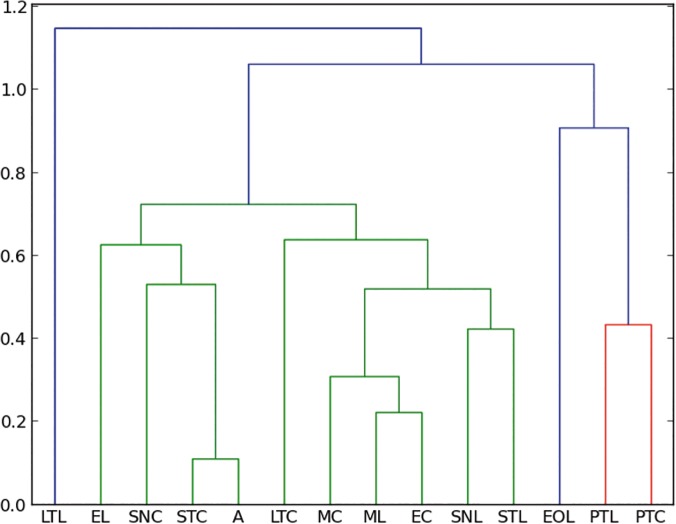
Manhattan–UPGMC cluster analysis of 25 orders sampled in the two collecting places “La Cadena” (L) and “Coquitos” (C), Chiapas, Mexico and in Mexican amber. ST Sticky trap, EO Eclector trap open, E Eclector trap, M Malaise trap, PT Pitfall trap, LT Light trap, SN Sweep netting, A Amber; modern sites with site specific suffix.

**Fig 5:**
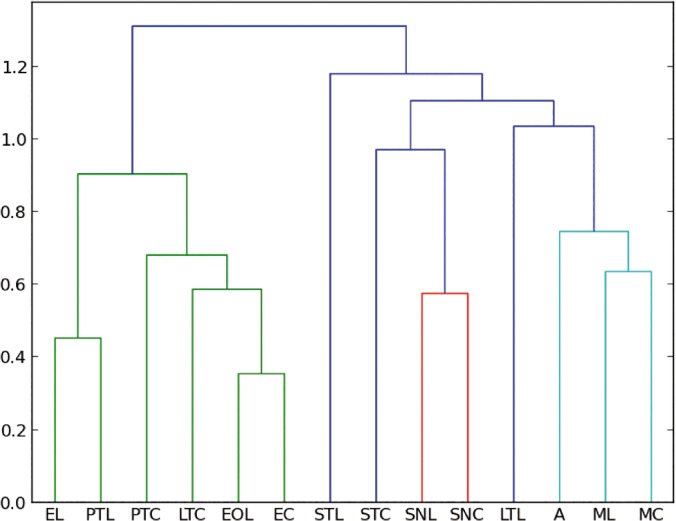
Manhattan—UPGMC cluster analysis of 24 families of Diptera sampled in the two collecting places “La Cadena” (L) and “Coquitos” (C), Chiapas, Mexico and in the Mexican amber. ST Sticky trap, EO Eclector trap open, E Eclector trap, Malaise, PT Pitfall, LT Light trap, SN Sweep netting, A Amber; modern sites with site specific suffix.

**Fig 6:**
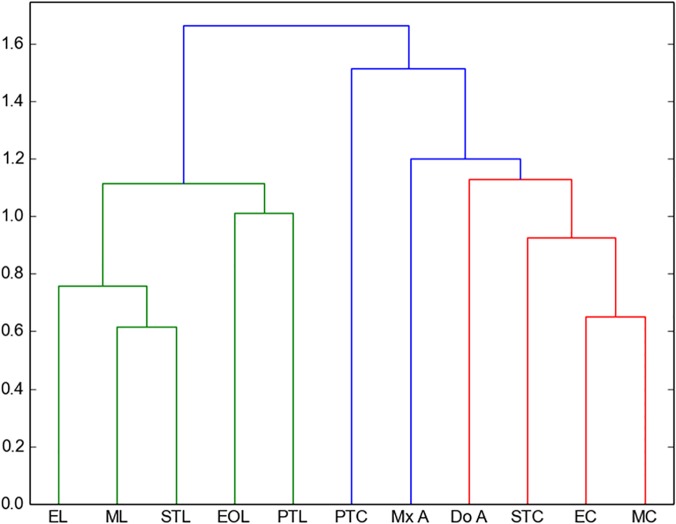
Manhattan–UPGMC cluster analysis of 54 families of spiders sampled in the two collecting places. **“La Cadena” (L) and “Coquitos” (C), Chiapas, Mexico.** ST Sticky trap, EO Eclector trap open, E Eclector trap, M Malaise trap, PT Pitfall trap, Mx–A Mexican amber, Do–A Dominican amber; modern sites with site specific suffix.

Despite the difference of size of the collections between Coquitos and La Cadena, with La Cadena providing almost twice the number of specimens in comparison with Coquitos, the percentage of most of the trapped orders and families is similar in both areas (Tables 1 and 2). Both collection places are to some degree dissimilar. Climatic conditions like humidity and temperature and floral composition are different (see Material and Methods– recent arthropods). These parameters play an important role for changes in the diversity of arthropods. *Hymenaea* for example was only found in the evergreen forest in La Cadena and aquatic Ephemeroptera and Trichoptera are clearly better represented in La Cadena (Table 1), where the humidity is higher than in Coquitos and a freshwater stream is running close to the *Hymenaea* trees. Some Diptera families like Mycetophilidae or Chironomidae, which prefer humid habitats, are also better represented in La Cadena than in Coquitos (Table 2). Our amber collections show that within all insects at the order level, Diptera at family level, and Arachnida at family level, good agreement exists with both Coquitos and La Cadena (Figs. 4, 5, 6).

**Table 1:** Total arthropods collected in Chiapas, Mexico with 7 different entomological traps in the rainy season as well as in the dry season.

Order	Sum of Count Coquitos	% of Total Coquitos	Sum of Count La Cadena	% of Total La Cadena
Diptera	7 378	45.715	16852	48.812
Hymenoptera	2 622	16.246	3748	10.856
Coleoptera	1 554	9.628	3201	9.271
Collembola	1 032	6.394	2991	8.663
Acari	849	5.260	2630	7.617
Lepidoptera	716	4.436	1092	3.163
Hemiptera: Auchenorrhyncha	576	3.568	1051	3.044
Aranea	488	3.023	730	2.114
Psocoptera	252	1.561	447	1.294
Heteroptera	180	1.115	400	1.158
Thysanoptera	136	0.842	373	1.080
Isoptera	127	0.786	333	0.964
Orthoptera	95	0.588	282	0.816
Blattodea	67	0.415	147	0.425
Hemiptera: Sternorrhyncha	31	0.192	115	0.333
Embidiina	12	0.074	49	0.141
Isopoda	9	0.055	21	0.060
Neuroptera	6	0.037	19	0.055
Pseudoscorpionida	5	0.030	18	0.052
Scorpionida	3	0.018	15	0.061
Ephemeroptera	1	0.006	4	0.011
Trichoptera	0	0.000	3	0.008
Mecoptera	0	0.000	2	0.005
Diplopoda	0	0.000	1	0.002
Chilopoda	0	0.000	0	0.000
TOTAL	16 139	100	34 524	100

**Table 2:** Total of Families of Diptera collected in “La Cadena” in Chiapas, Mexico with 7 different entomological traps in the rainy season as well as in the dry season.

Family	Sum of Count Coquitos	% of Total Coquitos	Sum of Count La Cadena	% of Total La Cadena
Culicidae	276	3.740	107	0.634
Ceratopogonidae	577	7.820	404	2.397
Chironomidae	307	4.161	1290	7.654
Psychodidae	464	6.288	631	3.744
Anisopodidae	0	0.000	1	0.005
Chaoboridae	0	0.000	2	0.011
Limoniidae	34	0.460	219	1.299
Tipulidae	0	0.000	6	0.035
Cecidomyiidae	952	12.903	5464	32.423
Sciaridae	414	5.611	3716	22.050
Keroplatidae	0	0.000	51	0.302
Mycetophilidae	29	0.393	422	2.504
Drosophilidae	2403	32.569	942	5.589
Muscidae	30	0.406	22	0.130
Asilidae	10	0.135	5	0.029
Phoridae	599	8.118	1899	11.268
Dolichopodidae	815	11.046	312	1.851
Tabanidae	46	0.623	2	0.011
Stratiomyidae	6	0.081	13	0.077
Scatopsidae	10	0.135	7	0.041
Tephritidae	0	0.000	11	0.065
Micropezidae	66	0.894	11	0.065
Empididae	50	0.677	46	0.272
Corethrellidae	0	0.000	3	0.017
Other Brachycera	285	3.862	494	2.931
Other Nematocera	5	0.067	424	2.516
Total	7 378	100	16 852	100

We collected in the dry and rainy season for three years in different months, so that the data can be interpreted as being representative of regional seasonality. A comparison at the order level between both seasons with the fauna included in amber was not informative, even if we analysed groups such as termites, which are indicative for a specific season and abundant in Mexican amber. According to Gomes da Silva Medeiros et al. [32] termites, synchronized flight occurs in different times of the year in Brazil, but the peak coincides with the onset of the rainy season. In our collection termites were clearly more abundant during the rainy period, principally at the beginning of the wet season during May. However, the total Mexican amber fauna appears to resemble a mixture of both rainy and dry season faunas. Seasonality was already evident during the Miocene [33] in the Amazonian forest and according to Jaramillo et al. [34] the climate in the lowland forest of the Neotropics was also similar to today. Resin production of *Hymenaea* trees depends of several genetic and environmental factors. The massive amount of Brazilian copal from Amazonia is often attributed to *H*. *courbaril* [35] but probably also derives from *H*. *oblongifolia* var. *palustris* (Ducke) Lee and Langenheim in periodically inundated areas. *Hymenaea* spp. that grow in moist forests or close to streams apparently synthesizes greatest quantities of resin, whereas species that live in dry forests produce less quantity of resin, but they produce relatively more during the wet season than in the dry season [36]. Thus, the amber from *H*. *mexicana*, the origin plant of Mexican amber [37], generally could have produced resin continuously, in wet and dry seasons.

The former Mexican amber forest has already been characterized as a lowland forest nearby mangroves [8, 18], and the Dominican amber forest as a lowland more interior neotropical rainforest [3]. However, the chironomids *Stenochironomus* and *Xestochironomus*, freshwater organism living in mountain streams have been described in Dominican amber [38]. Both the extent of the original amber forming forest and the distribution of *Hymenaea* during the Mexican Middle Miocene is unknown. *Hymenaea* was most probably disseminated by the sea, reaching the coast of South America and the south of Mexico during the early Cenozoic [39]. Today, some species of *Hymenaea* are found along the coast growing in sandy soils and drier ecosystems, while other species of *Hymenaea* grow mainly in evergreen forest ecosystems [39]. Most of the currently known Mexican amber has been derived from the area of Simojovel de Allende in the state of Chiapas, southern Mexico. However, more mines are appearing in other municipalities of Chiapas [9]. The amber from the Totolapa and Palenque regions is generally similar in faunal composition to that of the Simojovel area and they are probably of the same age [40, 41]. There are, however, some significant differences. Firstly, the level of preservation is particularly good, and secondly more than 7% of the totals are aquatic insects, like Ephemeroptera and Trichoptera, in comparison with the studied amber from Simojovel which contain about 2% aquatic insects.

These results underline the importance of a new analysis of the palaeoenvironment of the Mexican amber forest, compared with new amber material from new collection sites. Probably, the principal congregation of *Hymenaea* trees was within the lowland forest, close to the mangrove forest. However, the amber forest probably covered a wider area with conditions very similar to today in the Central and South American lowland forest.

### Which trap is the most similar to the amber?

With the collected arthropods from both collecting areas, we obtained dendrograms comparing all different types of traps with each other. No significant differences were found between different methods to produce the cluster (UPGMC, UPGM, and single linkage–clustering) as well as between different distance measures (Manhattan, and Euclidean), showing that the difference between traps are large enough to be detected with our data. As expected, both, Manhattan measure and Bray–Curtis index obtain exactly the same results. Fig. 4 shows the resulting dendrograms from a Manhattan–UPGMC cluster analysis of 25 orders sampled. Three distinct faunal groupings are evident in the dendrogram of the total of orders, not differentiating between the rainy or dry season. The trap method closest to the amber is, in this case, the sticky trap, followed by sweep netting, eclector and Malaise traps. Fig. 5 shows the resulting dendrogram of family level sampling within Diptera, in the two collecting sites. In this case, four distinct faunal groupings are evident and the amber is closely related first to Malaise traps and then to sweep netting and sticky traps. However, the position of the sweep netting has to be taken carefully, because only few specimens were trapped. There were less than 300 specimens compared to more than 1000 to 9000 in the other traps. The reason for this differences is the small amount of time dedicated to collection with sweep netting, especially during the rainy season, in comparison to the other traps, which remained all day and night for two days before being cleaned for the next two days.

Penney [15] used similar methods in a comparison of the spider fauna from Dominican amber with living spider faunas from a lowland deciduous rainforest in Panama and from a seasonal inundated forest in Peru. He reported that migrating spiders are more susceptible to entrapment than sedentary species. Furthermore, comparing sex ratio and feeding guilds, Penney [15] was able to confirm that active males are trapped more frequently. Penney [15] hypothesized that the resin that formed the amber deposits was secreted higher in the trunk region, farther away from the ground. As already mentioned above, these results contradicted the results of Henwood [14], who demonstrated that the amber fauna is most closely related to ground dwelling communities. Penney's results are based solely on the spider fauna and did not summarize the bias of all amber inclusions. In his work, canopy fogging, sweep netting and pitfall traps were compared with amber; our work does not include canopy fogging because it is a method that overestimates Aranea [42] and underestimates the number of Diptera, because Diptera often fly away from the fog, and fall down outside the collection sheets on the ground. However, in accordance with Penney [15], we conclude that the pitfall trap is not closely related to amber. Using Penney’s data from the Dominican amber, we compare also the living spiders from our traps with the Mexican and Dominican amber, excluding the traps with less than 50 specimens. The most frequent families within Archnida in both collections sites are Theriidae and Salticidae, well represented in the sticky traps and in amber (Table C in S1 File). A clustering analysis confirms the closeness of the Dominica amber to sticky traps (Fig. 6). However, the amount of data from Mexican amber is not comprehensive enough for a more accurate statistic and has to be tested in future works with more material.

If we take only families of Diptera into account, the trap most similar to amber is the Malaise trap (Figs. 5 and 6). Malaise and sticky traps do not catch arthropods in a similar way. Malaise is a kind of “aerial trap” intercepting most flying insects. Sticky traps intercept flying and migrating fauna on the trunk also capturing many crawling arthropods. In both Mexican and Dominican ambers, (similar in age and formational environment) Cecidomyiidae are the most abundant Diptera. Cecidomyiidae have also been collected with sticky traps in Mexico, but are not numerous. This is different from the results of the Malaise traps, where Cecidomyiidae are most frequent, particularly in La Cadena. In the case of Baltic amber, some of the most abundant Diptera are Chironomidae, Dolichopodidae and Mycetophylidae, followed by Sciaridae, according to the data of Larsson [10], and Chironomidae and Sciaridae according to Sontag [27]. Our data on Mexican amber differ from the data obtained by Sontag [27] from an unsorted sample in Baltic amber, but are very similar to the data obtained by Poinar and Poinar [7] from Dominican amber (same age ∼ 20 My and same sourse tree *Hymenaea*) from a sample of randomly selected pieces with inclusions used to analyze the frequency of organisms in this amber. The Sciaridae are the most abundant insects in the sticky traps. This has also been reported from other regions in the world: Bickel and Tasker [43] demonstrated the high frequency of Sciaridae trapped with sticky traps in Australia. On the other hand, Chironomidae was one of the most abundant families in the Malaise traps, probably because of trap proximity to streams. The distribution of ant subfamilies (Formicidae) from the collection sites and amber is shown in (Table D in S1 File). In amber the most frequent subfamilies are Dolichoderinae and Myrmicinae. Malaise, light traps and sticky traps seem to be similar to amber, however Malaise and light traps catch more winged ants than either the sticky trap or amber. Thus, at least for Diptera, Malaise traps have to be also taken in account if fossil and recent diversity are to be compared.

It can be summarized that amber or resin as an entomological trap corresponds to a combination of different artificial traps. An idealized artificial trap most similar to amber would consist of a mixture of the tested traps with the following percentages of insect orders: Open Eclector (1%), Malaise (7%), Pitfall (6%), Sweep netting (7%), and Sticky trap (79%). We obtained these data by performing a linear combination of the percentages of arthropods in different traps, and minimizing the distance from the percentages within the amber trap. With this method the similarity between amber and sticky trap at order level is confirmed. However, we also demonstrate here that amber or a resin trap cannot be compared with a single artificial trap; for comparisons of amber with recent fauna, several different traps must be taken into account.

Amber, or resin traps capture mostly small flying insects, similar to Malaise and sticky traps and sweep netting. All of these four traps principally catch Diptera. Arboral photo–eclectors trap a much larger percentage of non–winged arthropods living around the tree, such as collembolans and mites. Our light trap collected a large amount of macrolepidoptera, which are very rare in amber and other insects with positive phototaxis like aquatic insects, Ephemeroptera and Trichoptera [44]. Pitfalls obviously trap more arthropods that are exclusively soil inhabitants. However, pitfalls were included in the present study because some ambers have entrapped large numbers of soil arthropods [45] and Henwood [14] already hypothesized that amber reflects ground–subterranean level resin production. Our results indicate, as already suggested by Penney [15], that pitfall traps are dissimilar to amber. This is not only because amber collected more tree–inhabiting species but also because the pitfalls capture more than 40% apterous arthropods (Table 3), and more than 15% exclusive soil habitants.

**Table 3:** Differences in percent of the trapped arthropods with 7 trap methods.

	EO	E	M	LT	PT	ST	SN	Mx amber
winged	44.2%	75.0%	89.19%	68.73%	58.01%	98.83%	81.35%	88.45%
apterous	55.7%	25.0%	10.80%	31.26%	41.98%	10.16%	18.64%	11.57%
exclusive soil inhabitants	9.43%	1.53%	0.60%	0%	∼15%	0.69%	1.69%	0.17%
aquatic (adult form)	0%	∼0.46%	1.39%	1.19%	0%	0.14%	0.37%	2.26%
taxon majority	Acari	Diptera	Diptera	Acari	Collembola	Diptera	Diptera	Diptera
predators	10.36%	7.74%	2.60%	3.16%	4.54%	8.73%	13.44%	5.93%
scavenbgers	14.05%	11.64%	16.13%	5.10%	8.31%	15.42%	3.06%	4.55%
epiphyte grazers	0.68%	3.39%	1.67%	1.78%	0.91%	1.56%	2.59%	2.11%
sap suckers	9.14%	4.53%	5.55%	7.84%	6.52%	9.81%	11.55%	8.78%
tourists	24.82%	34.27%	23.64%	37.77%	23.65%	42.18%	39.38%	39.82%
chewers	22.91%	24.51%	40.56%	29.20%	1.71%	2.02%	9.90%	12.90%
parasitoids	11.32%	3.66%	2.00%	0.99%	1.79%	16.64%	10.84%	23.83%
ants	6.68%	10.23	7.81%	14.12%	41.09%	3.60%	9.19%	2.04%

EO: Eclector trap open, E: Eclector trap, M: Malaise trap, PT: Pitfall trap, LT: Light trap, SN: Sweep netting, ST: Sticky trap.

**Fig 7:**
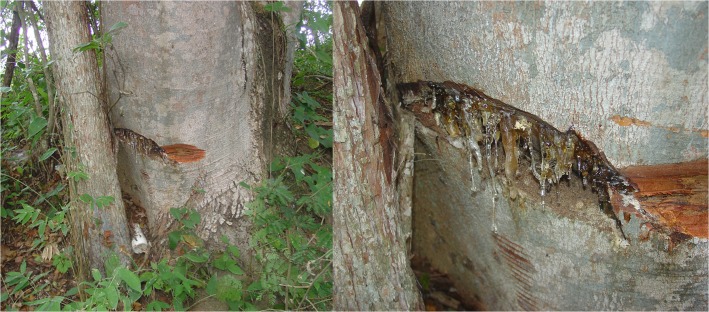
*Hymenaea courbaril* with resin production in Vallaflores, Chiapas, Mexico.

The results are based on the assumption that the communities of arthropods in the studied areas are similar to the communities in the former Miocene amber forest. This based, principally, on the similarity of the floral composition but also of the distribution of arthropods at different taxonomical level as well as the size distribution explained below. Ideally, we would include resin or copal (recently deposited resin) from *Hymenaea courbaril* for the comparison with amber to be completely sure that modern resin is trapping in a similar way to the amber, but unfortunately, *H*. *courbaril* produces only very small amounts of resin so that comparative statistical analyses are impracticable. Small amounts of resin were detected in Villaflores, Chiapas by Marco A. Coutiño José (Museo de Paleontología Eliseo Palacios in Tuxtla, Chiapas, Mexico) in one isolated *H*. *courbaril* tree (Fig. 7). However, the production began only about six months after a large lesion was slashed into the bark. The area in which this tree grows is strongly affected by human activities and ecologically not similar to the former Miocene Mexican amber forest. The resin collected from this lesion contained only very few inclusions, insufficient for any solid quantitative analysis. This observation confirms field observations and “slashing experiments” on living *H*. *courbaril* trees in Panama by Alison Henwood [46]. *Hymenaea* resin from other regions, e.g., Colombia or Madagascar, might help to corroborate the results of the present study, even if the ecological conditions and the fauna are completely different.

### Size and behavior of arthropods

**Fig 8:**
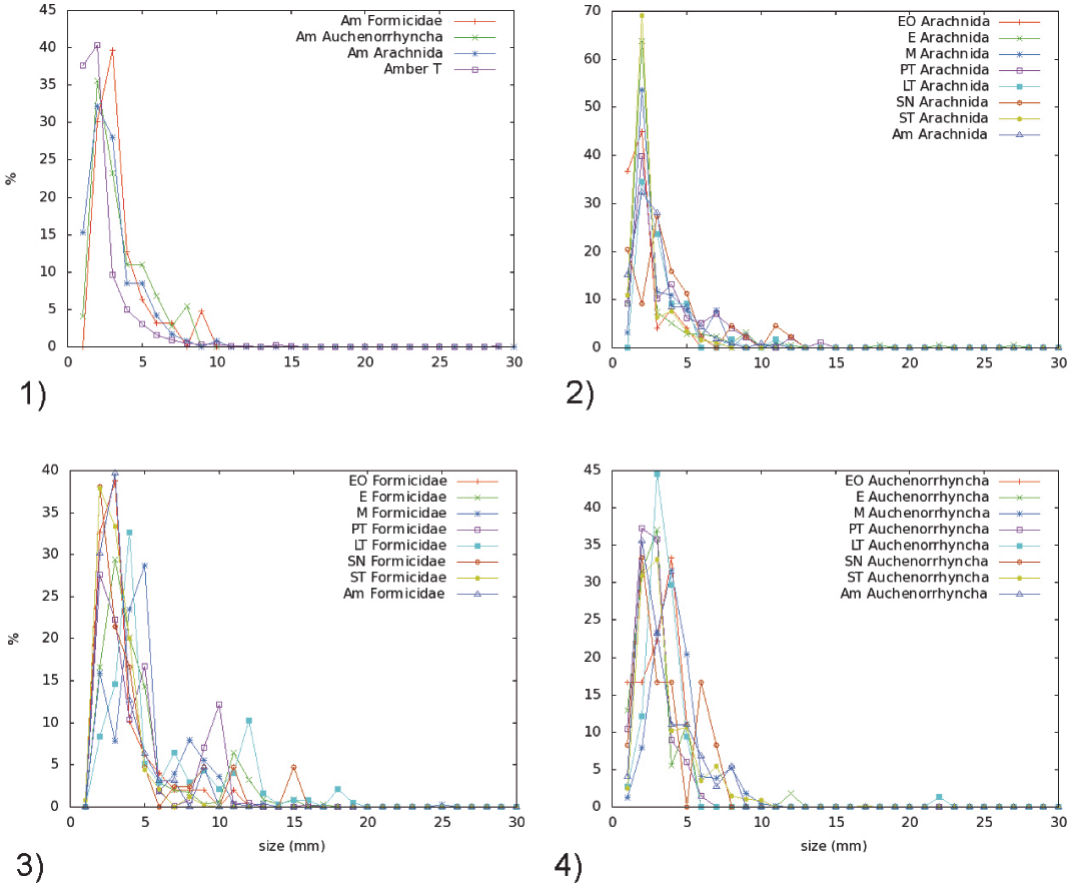
Distribution of body size. 1) Distribution of body size of all arthropods in amber (Amber T), all Arachnidae in amber (Am Arachnida), all Auchenorrhyncha in amber (Am Auchenorrhyncha), and all Formicidae in amber (Am Formicidae). 2) Distribution of body size of all Arachnidae in amber and in all traps in La Cadena and Coquitos in Chiapas, Mexico. 3) Distribution of body size of all Formicidae in amber and in all traps in La Cadena and Coquitos in Chiapas, Mexico. 4) Distribution of body size of all Auchenorrhyncha in amber and in all traps in La Cadena and Coquitos in Chiapas, Mexico. Am: Amber, EO Eclector trap open, E Eclector trap, M Malaise trap, PT Pitfall trap, LT Light trap, SN Sweep netting, ST Sticky trap.

The size of the arthropods in Mexican amber and some extant arthropod groups (Arachnida, Formicidae and Auchenorrhyncha) from all tested traps seems to have a skewed normal distribution of size. All of these traps display similar parameters instead of an exponential decay (Fig. 8), which was the initially expected distribution due to the bias of size. The same kind of distribution has been obtained qualitatively by Penney and Langan [17] for spiders, even with a smaller amount of data. Penney and Langan [17] concluded that the resin producing trees that created Baltic amber better favored large areal web–spinning than those that produced Dominican amber. The distribution of size in most of the families studied is positively skewed, showing that very small spiders were not the most frequent in amber. We obtained the same results from Mexican amber and can confirm that not only small spiders but other small arthropods are not the most frequently trapped inclusions in amber. This distribution is shown here not only in the study of the whole Mexican amber fauna, but also for the collection of living arthropods with seven different traps, obtaining always the same distribution pattern. This distribution seems to be the general shape of the species body size distribution in the animal kingdom [47]. We conclude that the size bias is qualitatively independent of the kind of trap for non–extreme values. The shape of the body size distribution depends of bias of trapping methods. However, the maximum of this distribution will stay more or less in the same position independently of the trapping method, as shown in Fig. 8. This peak is related to the ecosystem properties, obtaining the same for similar ecosystems [48]. We have observed that both Mexican amber arthropods and the collected extant arthropods present the same peak of body size distribution.

The studied collections of Mexican amber do not include arthropods larger than 10 mm. However, one exception has been published by Dunlop [49], a fossil tarantula, which represents the largest spider ever recorded from amber. In Dominican amber an elaterid beetle larger than 20 mm has been found (Keith Luzzi, AMNH New York, pers. comm.). The majority of specimens in Mexican amber are between 1 and 2 mm in body size, with the next most common group being between 0 and 1 mm. Very small springtails, thrips or mites are easily overlooked, however in the analyzed collections we played special attention to such small arthropods. They are frequently found in amber, but not as often as it would be expected based on our observations of trapping size bias.

The minor presence of Collembola in amber (in the case of Mexican amber, 3.36%), can be explained by their preferred habitat in leaf litter, although some species are abundant in rain forest canopies [50]. In all traps springtails were well preserved, with the exception of the sticky traps, where they remained exposed and without alcohol until they were removed from the sticky adhesive by a solvent. Our observations suggest that if the amber–forming resin had not covered the collembolans quickly, many of them would probably have decomposed. Another possible explanation is that Collembola land on and then just jump off the resin, without being captured. The best methods to collect Recent springtails were the Malaise and pitfall traps. In case of the mites, the light trap collected the largest number of specimens. The abundance of mites in Mexican amber, 2.5%, is not as high as might be expected, also in other unsorted amber collections (e.g. Spainish Cretaceous amber, 3.35%, Carmelo Corral, Museo de Ciencias Naturales de Alava, Spain pers. comm.). One potential explanation for the reduced numbers of mites could be that the major production of resin was not at ground level but higher up on the trunk. In contrast, the French amber contains 6.15% of mites (Vincent Perrichot, University of Rennes, France pers. Comm.), an amber deposit that is known to have entrapped large numbers of soil arthropods [45]. In the case of the Thysanoptera in Mexican amber (0.99%) it is well known that the monitoring of these small insects is especially successful with blue sticky traps, suggesting that color plays an important role, and may be a contributing factor in our sample comparison.

The percentage of winged insects in amber (88.45%) is generally similar to that obtained from sweep netting (81.35%), Malaise (89.19%) and sticky traps (98.83%). Within winged insects, the most common group in Mexican amber are the Diptera (37%), as also observed in sweep netting (42%), Malaise (64%), sticky (46%) and eclector (54%) traps. The most common families in Mexican and Dominican ambers are: Cecidomyiidae (37.33% and 19.58%), Phoridae (12.41% and 3.37%), Psychodidae (8.34% and 9.64%) Ceratopogonidae (5.90% and 8.05%), Chironomidae (5.90% and 8.84%). They are also the most common visitors among all Diptera on extant angiosperms [51] and within them; the high occurrence of Cecidomyiidae in *H*. *courbaril* var. *stignocarpa* is mentioned by dos Santos [52], which could be one of the reasons for their frequency in these ambers. Some species of the same families may appear in swarms facilitating the great number of entrapped specimens in resin and in aerial traps. The next largest group of flying insects in amber are the small parasitoid wasps (Hymenoptera) (23.83%), which are also well represented in the sticky traps (16.64%).

**Fig 9:**
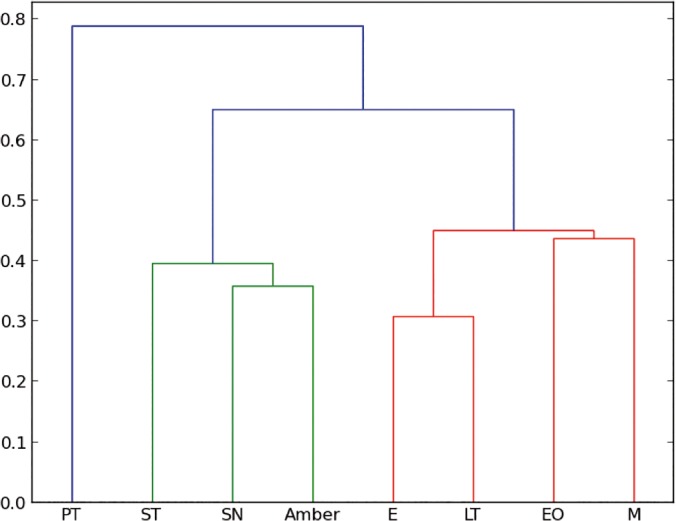
Manhattan–UPGMC cluster analysis of guilds sampled in the two collecting places “La Cadena” and “Coquitos”, Chiapas, Mexico and in the Mexican amber. PT Pitfall trap, ST Sticky trap, SN Sweep netting, E Eclector trap, LT Light trap, EO Eclector trap open, M Malaise trap.

The guild classification (Table 3) shows once more that the amber trap can be compared only with a mixture of artificial traps. In our collections, predators were well captured with open eclectors and sweep netting; scavengers with Malaise and sticky traps; epiphyte grazers with eclectors, sweep netting, and they are also frequent in amber; sap suckers with sweep netting and sticky traps; tourists (visitors) with sticky traps and frequent in amber, chewers with Malaise and light trap, parasitoids with sticky traps and amber and finally ants with light and pitfall traps. Surprisingly, using the guild classification for a clustering analysis the most closely related traps to amber are sweep netting and the sticky trap. However, the amount of data from the sweep netting was not comprehensive enough for a confident result (Fig. 9).

The knowledge of which trap is most closely related to the amber trap, together with information on the ecology of different taxa, is vital for the reliable and precise reconstruction of former ecosystems. We highlight this aspect with some specific examples:

#### Psychodidae

**Fig 10:**
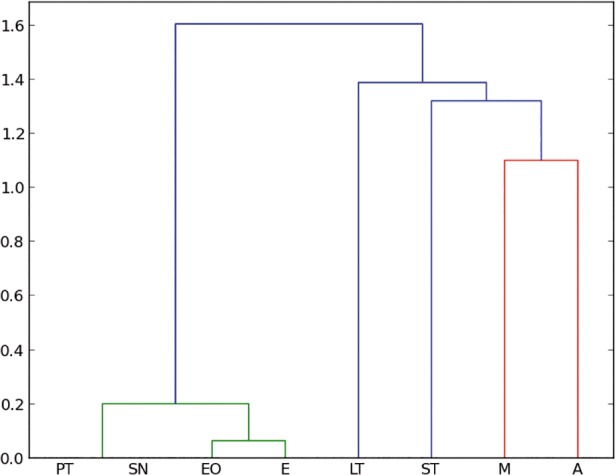
Manhattan–UPGMC cluster analysis of 19 genera of Psychodidae (Diptera) sampled in the two collecting places “La Cadena” and “Coquitos”, Chiapas, Mexico and in Mexican amber. ST Sticky trap, EO Eclector trap open, E Eclector trap, MC/ML Malaise trap, PT Pitfall trap, LT Light trap, A Amber.

This family is especially useful for comparison because they are moderately common in Mexican amber and have been intensively systematically studied [53–56]. The Psychodidae fauna from the Mexican amber compared with the trapped extant Psychodidae is represented in Fig. 10. The composition of the Psychodidae fauna trapped with malaise and sticky traps is most similar to the Psychodidae entombed in amber. Very interesting is the genus *Trichomyia* because it is represented in Mexican amber with 34 specimens (Table E in S1 File), the majority of which are females, from a total of 65 psychodid specimens. Species of *Trichomyia* inhabit dead wood and are thus likely to get trapped in resin. Some of the external mechanisms that can promote inducible production of significant amounts of resin are insects attack or pests, lesions or sickness. These factors can induce changes in the plant gene expressions that promote rapid increases in resin flows [57]. Possibly the trees or part of a tree inhabiting by *Trichomyia* produced more resin than completely healthy trees. Therefore, the abundance of *Trichomyia* in Mexican amber and other amber deposits [58, 59] in contrast to other genera of Psychodidae is interpreted as an ecological phenomenon rather than an unusual high diversity in the Mexican amber forest. Moreover, the dependency on dead wood, especially for breeding, would explain the large proportion of females in the investigated amber material [53]. The relatively poor representation of the genus in the artificial traps can be explained easily by the presence of few fallen trees in the modern forest, since these trees are of economic value, and because the collection of living arthropods has been carried out on healthy trees. Most of the living *Trichomyia* have been captured, for the present work, in the Malaise trap and not with the open eclector collocated on a fallen tree. This is probably because the Malaise is capturing more active flying insects, contrary to the open eclector.

#### Dolichopodidae

Dolichopodidae are one of the most common families in amber. They are well known from abundant Dominican amber inclusions, contrasting with the rather small number of Mexican amber inclusions. The most common Recent genus collected in Chiapas is *Chysotus*, mostly taken with sticky traps. It is not known from Mexican amber and is represented by only 5 specimens seen in the AMNH Dominican amber holdings (DJB, pers. obs.). The most common genus in Dominican amber is *Medetera* (Table F in S1 File), a cosmopolitan genus today found resting on tree trunks [60]. The reason for its high frequency in amber, as in case of *Trichomyia*, is here interpreted as an ecological effect rather than an unusual abundance in the former Mexican amber forest.

#### Formicidae

Table D in S1 File shows the distribution of subfamilies of Formicidae from the artificial traps and in amber. The majority of recent specimens collected belong to the subfamilies Myrmicinae and Ponerinae. In amber the most common subfamilies are Dolichoderinae and Myrmicinae, as well as for the sticky traps, with the dolichoderine genus *Azteca* being the most common. The genus *Azteca* has a neotropical distribution, with many species, all of which are arboreal [61], which explains their great abundance in amber.

The exemplary analyses at the subfamily and genus level clearly show that frequent specimens in amber were not necessarily the most frequent arthropods in the former amber forest. Selected species with higher numbers of specimens appear in amber because of their ecology and behavior, usually closely related with a tree–inhabiting life.

We conclude that at the insect ordinal level, sticky traps are most similar trap to Mexican and Dominican ambers. However, Malaise trap and sweep netting must also be taken into account in such a comparison. Changes of diversity from the Middle Miocene to Recent time in Central and South America can be analyzed by comparing the rich amber faunas from Mexico and Dominican Republic and copal from Costa Rica or Colombia with the fauna trapped with sticky and Malaise traps, and sweep netting in the lowland forests from the same regions. Sticky traps are more commonly used for monitoring pest populations, however the present work and the study of Bickel and Tasker [43] demonstrate that sticky traps can be also useful for the analysis of the tree trunk arthropod diversity of a specific region.

### Was the diversity during the Miocene higher than today?

**Fig 11:**
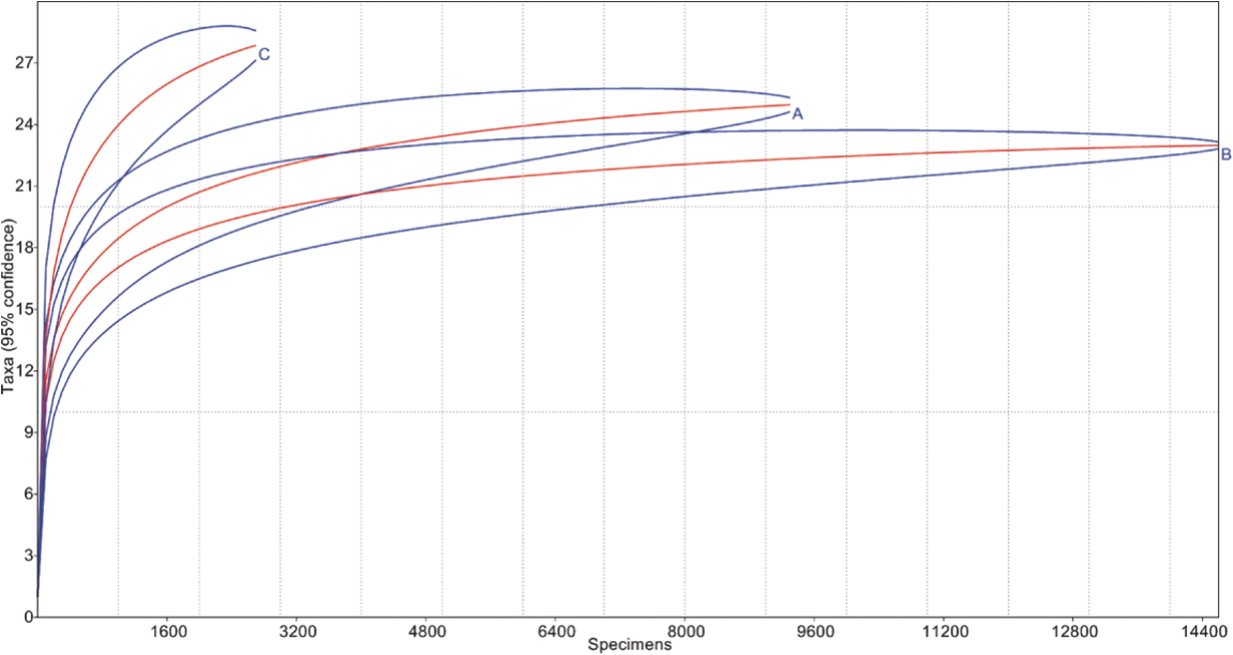
Rarefaction analysis. Where A represent Sticky traps, B the Malaise traps and C the amber (resin trap).

**Fig 12:**
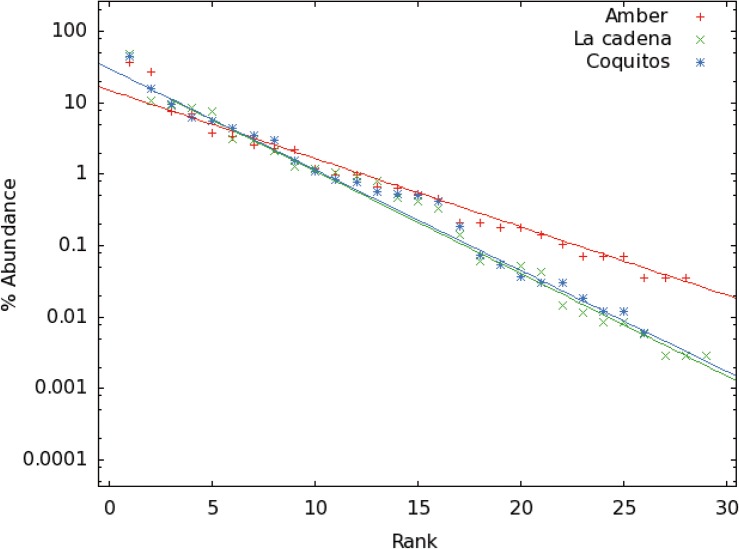
Exponential distribution of probabilities of both collecting places (La Cadena and Coquitos) and amber fauna. The three continous lines represent exponential functions A*exp(–b x) with parameters A and b that fits to the data (points) obtained in the collections. A = 0.3 for the collection in Coquitos and La Cadena, A = 0.15 for amber, and b = 0.33, b = 0.325, and b = 0.22 for La Cadena, Coquitos and amber respectively.

Surprisingly, the rarefaction analysis (Fig. 11) show that the fossil amber trap has a higher diversity in comparison with Malaise and sticky traps, which are the traps with the largest amount of data. Fig. 12 shows that both La Cadena and Coquitos are less diverse in comparison to the amber. Was the diversity during the Miocene higher than today? Or is this high diversity in amber biased by factors like the overall longer period of collection? Long term collections are necessary for the study of biodiversity because, long term trapping produces a collection that takes the dynamics of the ecosystem (eg. wild fires, climate variability) or the natural variability of ecosystems into account [62]. Long term collections have the possibility to catch rarely observed arthropods with a long diapause, or univoltine insects. In contrast, short term collections do not collect arthropods with life cycles longer than the collecting window or with a very short life time as adult. Furthermore, resin as an entomological trap, even if it is biased, is a very effective trap, offering the possibility to catch arthropods not only on the tree trunk and branches, but also on the roots and leaf litter. Also important is the long time period during which the resin can be sticky, so that even rare or unusual faunal elements will be eventually trapped. Perhaps the most important driver for increased diversity in amber deposits is that a single amber deposit represents an incalculable time period of sampling.

Mexican amber belongs to the Middle Miocene, a time of climate optimum and a time of high diversification in the terrestrial realm because of change of atmospheric CO_2_ [63]. Tappert et al [64] considered that the CO_2_ level during the Middle Miocene was higher than at present. Since high atmospheric values have been correlated with greenhouse warming and with high diversity of insects during the Eocene (e.g in South America [65]), higher levels of CO_2_ could have a similar effect during the Miocene. However, a dry forest ecosystem had already been established by the Middle Miocene in southern Mexico [66], and the climate in the lowland of the Neotropics during the Middle Miocene appears to be similar to modern conditions, even if this assumption is controversial [34]. Looking more precisely at Tables E and F in S1 File, the occurrence of genera of Psychodidae and Dolichopodidae in Mexican and Dominican ambers seems to be similar to the modern Neotropical forest in Central America, even within groups highly sensitive to changes like aquatic or semiaquatic dipterans. The lack of some genera in amber could be the result of lack of data; however we cannot reject the possibility that these genera were absent in Central America during the Middle Miocene. Thus, if diversity of arthropods during the Miocene was higher or lower than today is a still open discussion, and quantitative analyses of representative samples of species in amber could contribute to gaining insight into this challenging question.

## Supporting Information

S1 File Total of specimens of the different traps used to collect in *Bursera simaruba* within the Biosphere reserve “La Encrucijada”and in *Hymenaea courbaril* on the edges of the Biosphere reserve “La Encrucijada” and in Mexican and Dominican ambers. (DOCX)
