# Correction: Entrapment Bias of Arthropods in Miocene Amber Revealed by Trapping Experiments in a Tropical Forest in Chiapas, Mexico

**DOI:** 10.1371/journal.pone.0126046

**Published:** 2015-05-06

**Authors:** The PLOS ONE Staff

## Notice of Republication

This article was republished on April 1, 2015, to correct an error in the citation: Solórzano Kraemer MM was incorrectly listed as Kraemer MMS. The publisher apologizes for the error. Please download this article again to view the correct version.
