# Supplementary material for: Entrapment Bias of Arthropods in Miocene Amber Revealed by Trapping Experiments in a Tropical Forest in Chiapas, Mexico
Source: PLoS One. 2015 Mar 18;10(3):e0118820. doi: 10.1371/journal.pone.0118820 (PMC4364730; doi:10.1371/journal.pone.0118820)
Supplement: S1 File — (DOCX) [file pone.0118820.s001.docx]

**File S1**

**Supporting Information**

**Table A. Total of specimens of the different traps used to collect in *Bursera simaruba* within the Biosphere reserve “La Encrucijada” in the collection area “Coquitos” (C) and in *Hymenaea courbaril* on the edges of the Biosphere reserve “La Encrucijada” in the collection area “La Cadena” (L) and in Mexican amber.** EO: Eclector trap open, E: Eclector trap, M: Malaise trap, PT: Pitfall trap, LT: Light trap, SN: Sweep netting, ST: Sticky trap.

| **Order** | **EOC** | **EC** | **MC** | **LTC** | **PTC** | **STC** | **SNC** | **EOL** | **EL** | **ML** | **LTL** | **PTL** | **STL** | **SNL** | **Mx AMBER** |
| --- | --- | --- | --- | --- | --- | --- | --- | --- | --- | --- | --- | --- | --- | --- | --- |
| Acari | 338 | 91 | 188 | 21 | 74 | 180 | 17 | 241 | 279 | 209 | 1828 | 134 | 298 | 2 | 73 |
| Aranae | 18 | 153 | 55 | 20 | 32 | 171 | 39 | 47 | 78 | 97 | 34 | 71 | 111 | 9 | 108 |
| Hemiptera:Auchenorrhyncha | 0 | 36 | 43 | 26 | 25 | 445 | 1 | 18 | 19 | 583 | 47 | 41 | 372 | 12 | 200 |
| Blattodea | 0 | 15 | 7 | 5 | 10 | 20 | 10 | 0 | 6 | 11 | 2 | 2 | 28 | 0 | 13 |
| Chilopoda | 0 | 0 | 0 | 0 | 0 | 0 | 0 | 0 | 0 | 0 | 0 | 2 | 0 | 0 | 3 |
| Coleoptera | 19 | 317 | 66 | 329 | 249 | 557 | 17 | 150 | 185 | 667 | 659 | 324 | 631 | 20 | 215 |
| Collembola | 52 | 408 | 20 | 8 | 242 | 298 | 4 | 460 | 529 | 992 | 27 | 724 | 464 | 5 | 95 |
| Diplopoda | 0 | 0 | 0 | 0 | 0 | 0 | 0 | 0 | 0 | 0 | 0 | 0 | 3 | 0 | 6 |
| Diptera | 23 | 2691 | 1110 | 1140 | 31 | 2304 | 79 | 550 | 866 | 9518 | 575 | 215 | 4980 | 148 | 1041 |
| Embioidea | 0 | 3 | 0 | 3 | 0 | 6 | 0 | 0 | 0 | 0 | 5 | 0 | 10 | 0 | 0 |
| Ephemeroptera | 0 | 0 | 0 | 0 | 0 | 0 | 0 | 0 | 0 | 4 | 15 | 0 | 2 | 0 | 18 |
| Heteroptera | 2 | 54 | 22 | 4 | 45 | 50 | 3 | 8 | 7 | 61 | 71 | 14 | 89 | 32 | 28 |
| Hymenoptera | 20 | 271 | 149 | 95 | 306 | 1735 | 46 | 111 | 208 | 1162 | 310 | 541 | 1369 | 47 | 772 |
| Isopoda | 8 | 1 | 1 | 0 | 5 | 2 | 0 | 0 | 0 | 0 | 0 | 0 | 0 | 0 | 5 |
| Isoptera | 0 | 0 | 0 | 34 | 2 | 90 | 1 | 7 | 5 | 16 | 100 | 3 | 599 | 0 | 65 |
| Lepidoptera | 0 | 58 | 124 | 491 | 2 | 37 | 4 | 4 | 14 | 797 | 215 | 1 | 16 | 4 | 15 |
| Mecoptera | 0 | 0 | 0 | 0 | 0 | 0 | 0 | 0 | 0 | 1 | 0 | 0 | 0 | 0 | 0 |
| Neuroptera | 0 | 1 | 1 | 0 | 4 | 0 | 0 | 1 | 0 | 8 | 2 | 0 | 5 | 3 | 1 |
| Orthoptera | 0 | 30 | 7 | 1 | 38 | 14 | 5 | 8 | 19 | 210 | 1 | 63 | 24 | 8 | 19 |
| Pseudoscorpionida | 0 | 0 | 1 | 0 | 3 | 0 | 0 | 4 | 9 | 0 | 1 | 2 | 2 | 0 | 5 |
| Psocoptera | 0 | 54 | 36 | 6 | 3 | 144 | 9 | 5 | 59 | 196 | 41 | 21 | 82 | 2 | 63 |
| Scorpionida | 2 | 1 | 0 | 0 | 0 | 0 | 0 | 2 | 0 | 0 | 0 | 0 | 0 | 0 | 1 |
| Hemiptera: Sternorrhyncha | 0 | 1 | 2 | 1 | 2 | 25 | 0 | 5 | 2 | 26 | 14 | 11 | 89 | 0 | 6 |
| Thysanoptera | 5 | 9 | 14 | 4 | 4 | 98 | 2 | 11 | 20 | 20 | 39 | 29 | 254 | 0 | 28 |
| Trichoptera | 0 | 0 | 0 | 0 | 0 | 0 | 0 | 0 | 0 | 44 | 59 | 0 | 10 | 2 | 33 |

**Table B. Total of Diptera specimens of the different traps used to collect in *Bursera simaruba* within the Biosphere reserve “La Encrucijada” in the collection area “Coquitos” (C) and in *Hymenaea courbaril* on the edges of the Biosphere reserve “La Encrucijada” in the collection area “La Cadena” (L) and in Mexican amber.** EO: Eclector trap open, E: Eclector trap, M: Malaise trap, PT: Pitfall trap, LT: Light trap, SN: Sweep netting, ST: Sticky trap.

| **Family** | **EOC** | **EC** | **MC** | **LTC** | **PTC** | **STC** | **SNC** | **EOL** | **EL** | **ML** | **LTL** | **PTL** | **STL** | **SNL** | **Mx AMBER** |
| --- | --- | --- | --- | --- | --- | --- | --- | --- | --- | --- | --- | --- | --- | --- | --- |
| Anisopodidae | 0 | 0 | 0 | 0 | 0 | 0 | 0 | 0 | 1 | 0 | 0 | 0 | 0 | 0 | 1 |
| Asilidae | 0 | 4 | 5 | 0 | 0 | 0 | 1 | 0 | 0 | 1 | 0 | 1 | 3 | 2 | 2 |
| Cecidomyiidae | 5 | 470 | 319 | 0 | 17 | 128 | 13 | 159 | 275 | 4886 | 42 | 44 | 112 | 21 | 370 |
| Ceratopogonidae | 2 | 110 | 33 | 0 | 186 | 238 | 8 |  | 38 | 275 | 4 | 41 | 31 | 15 | 63 |
| Chaoboridae | 0 | 0 | 0 | 0 | 0 | 0 | 0 | 0 | 2 | 0 | 0 | 0 | 0 | 0 | 0 |
| Chironomidae | 1 | 7 | 66 | 0 | 7 | 215 | 11 | 1 | 23 | 844 | 3 | 99 | 315 | 5 | 62 |
| Corethrelidae | 0 | 0 | 0 | 0 | 0 | 0 | 0 | 0 | 00 | 2 | 0 | 0 | 0 | 0 | 0 |
| Culicidae | 0 | 16 | 174 | 0 | 53 | 23 | 10 | 1 | 5 | 52 | 0 | 1 | 0 | 48 | 13 |
| Dolichopodidae | 3 | 5 | 35 | 4 | 24 | 741 | 3 | 3 | 8 | 158 | 1 | 0 | 137 | 5 | 46 |
| Drosophilidae | 0 | 1622 | 38 | 18 | 716 | 6 | 3 | 298 | 291 | 196 | 72 | 128 | 40 | 17 | 7 |
| Empididae | 0 | 0 | 1 | 0 | 0 | 49 | 0 | 0 | 0 | 1 | 0 | 1 | 44 | 0 | 12 |
| Keroplatidae | 0 | 0 | 0 | 0 | 0 | 0 | 0 | 4 | 12 | 35 | 0 | 0 | 0 | 0 | 4 |
| Limoniidae | 0 | 1 | 20 | 4 | 5 | 3 | 1 | 0 | 8 | 202 | 0 | 7 | 2 | 0 | 2 |
| Micropezidae | 0 | 0 | 24 | 0 | 0 | 42 | 0 | 0 | 0 | 5 | 0 | 0 | 6 | 0 | 0 |
| Muscidae | 0 | 1 | 9 | 1 | 0 | 19 | 0 | 0 | 1 | 15 | 5 | 0 | 1 | 0 | 0 |
| Mycetophilidae | 0 | 7 | 18 | 0 | 0 | 4 | 0 | 9 | 9 | 391 | 2 | 0 | 11 | 0 | 29 |
| Phoridae | 1 | 85 | 26 | 2 | 9 | 460 | 16 | 20 | 109 | 75 | 53 | 30 | 1597 | 1302 | 166 |
| Psychodidae | 5 | 273 | 31 | 1 | 98 | 56 | 0 | 20 | 4 | 498 | 0 | 56 | 51 | 2 | 82 |
| Scatopsidae | 0 | 0 | 0 | 0 | 0 | 10 | 0 | 0 | 1 | 1 | 0 | 1 | 4 | 0 | 10 |
| Sciaridae | 1 | 35 | 230 | 0 | 2 | 144 | 2 | 13 | 56 | 1636 | 10 | 125 | 1876 | 1 | 62 |
| Stratiomyidae | 0 | 0 | 6 | 0 | 0 | 0 | 0 | 0 | 0 | 12 | 0 | 1 | 0 | 0 | 0 |
| Tabanidae | 0 | 14 | 32 | 0 | 0 | 0 | 0 | 1 | 0 | 1 | 0 | 0 | 0 | 0 | 3 |
| Tephritidae | 0 | 0 | 0 | 0 | 0 | 0 | 0 | 0 | 0 | 2 | 0 | 0 | 9 | 0 | 0 |
| Tipulidae | 0 | 0 | 0 | 0 | 0 | 0 | 0 | 1 | 0 | 4 | 1 | 0 | 0 | 0 | 7 |
| Other Brachycera | 2 | 41 | 43 | 1 | 23 | 164 | 11 | 20 | 20 | 155 | 22 | 39 | 387 | 19 | 46 |
| Other Nematocera | 3 | 0 | 0 | 0 | 0 | 2 | 0 | 0 | 6 | 67 | 0 | 1 | 354 | 0 | 54 |

**Table C. Total of Arachnida specimens of the different traps used to collect in *Bursera simaruba* and in *Hymenaea courbaril* and in Mexican and Dominican ambers*.*** EO: Eclector trap open, E: Eclector trap, M: Malaise trap, PT: Pitfall trap, LT: Light trap, SN: Sweep netting, ST: Sticky trap. Mx: Mexico. Do: Dominic Republic.

| Arachnida famlies | EO | E | LT | PT | M | ST | SN | Mx Amber | Do amber |
| --- | --- | --- | --- | --- | --- | --- | --- | --- | --- |
| Anyphaenidae | 0 | 1 | 0 | 0 | 2 | 2 | 0 | 0 | 16 |
| Agelenidae | 0 | 1 | 0 | 0 | 0 | 0 | 0 | 0 | 2 |
| Anapidae | 0 | 0 | 0 | 0 | 0 | 0 | 0 | 0 | 2 |
| Araneidae | 0 | 0 | 0 | 0 | 2 | 18 | 7 | 2 | 37 |
| Argiopidae | 0 | 0 | 0 | 0 | 0 | 0 | 0 | 1 | 0 |
| Barychelidae | 0 | 0 | 0 | 0 | 0 | 0 | 0 | 0 | 3 |
| Caponiidae | 2 | 5 | 0 | 0 | 0 | 0 | 0 | 0 | 4 |
| Clubionidae | 2 | 0 | 6 | 0 | 15 | 1 | 0 | 2 | 6 |
| Corinnidae | 1 | 16 | 2 | 6 | 6 | 6 | 0 | 0 | 25 |
| Ctenidae | 2 | 5 | 1 | 3 | 1 | 1 | 2 | 0 | 6 |
| Cyrtaucheniidae | 0 | 0 | 0 | 0 | 0 | 0 | 0 | 0 | 1 |
| Cybaeidae | 0 | 0 | 0 | 2 | 0 | 0 | 0 | 0 | 0 |
| Deinopidae | 0 | 1 | 0 | 0 | 0 | 0 | 0 | 0 | 0 |
| Dictynidae | 1 | 0 | 4 | 1 | 7 | 4 | 1 | 0 | 32 |
| Dipluridae | 0 | 1 | 1 | 0 | 1 | 0 | 0 | 0 | 5 |
| Dysderidae | 0 | 0 | 0 | 0 | 0 | 0 | 0 | 2 | 0 |
| Gnaphosidae | 0 | 2 | 0 | 1 | 2 | 1 | 0 | 1 | 8 |
| Hahniidae | 0 | 0 | 0 | 2 | 0 | 0 | 0 | 0 | 1 |
| Hersiliidae | 0 | 0 | 0 | 0 | 0 | 1 | 0 | 5 | 10 |
| Idiopidae | 0 | 0 | 0 | 2 | 0 | 0 | 0 | 0 | 0 |
| Linyphiidae | 7 | 2 | 0 | 2 | 1 | 9 | 0 | 1 | 13 |
| Liocranidae | 2 | 0 | 0 | 0 | 1 | 0 | 0 | 2 | 1 |
| Lycosidae | 0 | 1 | 0 | 27 | 1 | 0 | 0 | 0 | 1 |
| Micristigmatidae | 0 | 0 | 0 | 0 | 0 | 0 | 0 | 0 | 1 |
| Mimetidae | 0 | 0 | 0 | 1 | 0 | 0 | 8 | 0 | 9 |
| Miturgidae | 0 | 0 | 0 | 0 | 0 | 0 | 0 | 1 | 2 |
| Mygalomorphae | 1 | 0 | 0 | 1 | 0 | 0 | 0 | 0 | 0 |
| Mysmenidae | 0 | 0 | 0 | 0 | 0 | 0 | 0 | 1 | 2 |
| Nemesiidae | 0 | 1 | 0 | 0 | 0 | 0 | 0 | 0 | 1 |
| Nesticidae | 0 | 9 | 0 | 0 | 0 | 0 | 0 | 0 | 1 |
| Ochyroceratidae | 0 | 0 | 0 | 0 | 0 | 0 | 0 | 0 | 4 |
| Oecobiidae | 0 | 0 | 0 | 0 | 0 | 0 | 0 | 0 | 4 |
| Oonopidae | 6 | 8 | 1 | 7 | 2 | 4 | 0 | 0 | 16 |
| Oxyopidae | 0 | 1 | 0 | 2 | 4 | 0 | 0 | 1 | 8 |
| Palpimanidae | 0 | 0 | 0 | 0 | 0 | 0 | 0 | 0 | 4 |
| Philodromidae | 0 | 0 | 0 | 0 | 1 | 0 | 2 | 0 | 4 |
| Pholcidae | 6 | 48 | 5 | 8 | 13 | 25 | 3 | 1 | 35 |
| Pisauridae | 0 | 0 | 3 | 1 | 7 | 0 | 0 | 0 | 1 |
| Salticidae | 8 | 12 | 14 | 11 | 19 | 35 | 4 | 5 | 107 |
| Scytodidae | 1 | 16 | 0 | 2 | 6 | 4 | 3 | 0 | 8 |
| Segestriidae | 0 | 1 | 0 | 0 | 0 | 0 | 0 | 0 | 2 |
| Selenopidae | 0 | 3 | 0 | 0 | 3 | 2 | 0 | 1 | 8 |
| Sicariidae | 0 | 0 | 0 | 0 | 0 | 0 | 0 | 0 | 11 |
| Sparassidae | 0 | 0 | 0 | 0 | 2 | 2 | 0 | 3 | 1 |
| Synotaxidae | 0 | 0 | 0 | 0 | 0 | 0 | 0 | 1 | 0 |
| Tetrablemmidae | 0 | 0 | 0 | 0 | 0 | 0 | 0 | 0 | 1 |
| Tetragnathidae | 0 | 0 | 0 | 1 | 1 | 1 | 1 | 0 | 56 |
| Theraphosidae | 0 | 1 | 0 | 0 | 0 | 0 | 0 | 4 | 1 |
| Theridiidae | 7 | 64 | 15 | 7 | 43 | 70 | 5 | 7 | 222 |
| Theridiosomatidae | 0 | 0 | 0 | 0 | 0 | 0 | 0 | 0 | 4 |
| Thomisisdae | 0 | 0 | 0 | 0 | 2 | 0 | 0 | 1 | 5 |
| Trochanteriidae | 0 | 0 | 0 | 0 | 0 | 0 | 0 | 1 | ? |
| Uloboridae | 0 | 0 | 0 | 0 | 0 | 3 | 0 | 1 | 10 |
| Zodariidae | 0 | 0 | 0 | 0 | 0 | 0 | 0 | 1 | 0 |

**Table D. Total of Formicidae specimens of the different traps used to collect in *Bursera simaruba* and in *Hymenaea courbaril.*** EO: Eclector trap open, E: Eclector trap, M: Malaise trap, PT: Pitfall trap, LT: Light trap, SN: Sweep netting, ST: Sticky trap, Mx: Mexico. Do: Dominic Republic, n-w: wingless, w: winged.

| Formicinae | EO |  | E |  | M |  | LT |  | PT |  | ST |  | SN |  | Mx Amber |  |
| --- | --- | --- | --- | --- | --- | --- | --- | --- | --- | --- | --- | --- | --- | --- | --- | --- |
|  | n-w | w | n-w | w | n-w | w | n-w | w | n-w | w | n-w | w | n-w | w | n-w | w |
| Cerapachyinae | 0 | 0 | 0 | 0 | 0 | 37 | 0 | 0 | 0 | 0 | 0 | 0 | 0 | 0 | 0 | 0 |
| Dolichoderinae | 1 | 0 | 10 | 0 | 32 | 146 | 60 | 3 | 16 | 2 | 49 | 23 | 6 | 0 | 19 | 0 |
| Ecitoninae | 0 | 0 | 0 | 0 | 1 | 17 | 0 | 33 | 25 | 0 | 0 | 2 | 0 | 0 | 0 | 0 |
| Formicinae | 8 | 1 | 35 | 1 | 102 | 28 | 19 | 46 | 25 | 0 | 33 | 40 | 14 | 1 | 2 | 3 |
| Myrmicinae | 31 | 2 | 189 | 7 | 151 | 96 | 39 | 129 | 387 | 6 | 157 | 96 | 13 | 0 | 16 | 1 |
| Ponerinae | 3 | 2 | 32 | 1 | 17 | 290 | 0 | 39 | 293 | 6 | 8 | 81 | 3 | 1 | 5 | 3 |
| Pseudomyrmicinae | 1 | 0 | 68 | 0 | 21 | 3 | 1 | 2 | 1 | 0 | 24 | 1 | 1 | 0 | 0 | 0 |
| unidentified Formicidae | 0 | 0 | 0 | 0 | 0 | 0 | 0 | 0 | 0 | 0 | 7 | 1 | 0 | 0 | 3 | 6 |

**Table E. Total of Psychodidae specimens in amber and in the different traps*.*** EO: Eclector trap open, E: Eclector trap, M: Malaise trap, PT: Pitfall trap, LT: Light trap, SN: Sweep netting, ST: Sticky trap, Mx: Mexico. Do: Dominic Republic.

| Psychodidae | EO | E | M | PT | LT | SN | ST | Mx amber | Do amber |
| --- | --- | --- | --- | --- | --- | --- | --- | --- | --- |
| *Alepia* | 0 | 0 | 48 | 0 | 0 | 0 | 4 | 1 | 1 |
| *Arisemus* | 0 | 1 | 41 | 0 | 3 | 0 | 1 | 0 | 0 |
| *Australopericoma* | 0 | 0 | 20 | 0 | 71 | 0 | 1 | 1 | 0 |
| *Brunettia* | 0 | 0 | 13 | 0 | 0 | 0 | 0 | 1 | 1 |
| *Clogmia* | 0 | 0 | 1 | 0 | 1 | 0 | 0 | 0 | 0 |
| *Duckhousiella* | 0 | 0 | 2 | 0 | 0 | 0 | 0 | 0 | 0 |
| *Feuerborniella* | 0 | 0 | 10 | 0 | 2 | 0 | 0 | 0 | 0 |
| *Lepidiella* | 0 | 0 | 58 | 0 | 0 | 0 | 8 | 0 | 0 |
| *Lutzomyia* | 1 | 18 | 10 | 0 | 0 | 0 | 45 | 1 | 11 |
| *Maruina* | 0 | 0 | 73 | 0 | 0 | 0 | 2 | 0 | 0 |
| *Matuna* *(extinct)* | 0 | 0 | 0 | 0 | 0 | 0 | 0 | 2 | 0 |
| *Nemopalpus* | 0 | 0 | 0 | 0 | 0 | 0 | 0 | 0 | 1 |
| *Pericoma* | 0 | 0 | 2 | 0 | 0 | 0 | 0 | 0 | 0 |
| *Pintomyia* | 0 | 0 | 0 | 0 | 0 | 0 | 0 | 1 | 1 |
| *Philosepedon* | 1 | 20 | 81 | 0 | 4 | 0 | 0 | 6 | 1 |
| *Psychoda* | 26 | 432 | 87 | 3 | 27 | 2 | 16 | 8 | 6 |
| *Quatiela* | 0 | 0 | 0 | 0 | 0 | 0 | 0 | 0 | 1 |
| *Succinarisemus (extinct)* | 0 | 0 | 0 | 0 | 0 | 0 | 0 | 1 | 1 |
| *Telmatoscopus* | 0 | 0 | 1 | 0 | 2 | 0 | 0 | 1 | 0 |
| *Threticus* | 0 | 0 | 1 | 0 | 0 | 0 | 0 | 3 | 1 |
| *Trichomyia* | 0 | 0 | 38 | 0 | 8 | 0 | 3 | 34 | 11 |
| *Trilchopygomyia (extinct)* | 0 | 0 | 0 | 0 | 0 | 0 | 0 | 0 | 1 |
| Unidentified | 0 | 5 | 0 | 0 | 6 | 0 | 8 | 0 | 27 |

**Table F. Total of Dolichopodidae specimens in amber and in the different traps used to collect in *Bursera simaruba* and in *Hymenaea courbaril.*** PT: Pitfall trap, EO: Eclector trap open, E: Eclector trap, M: Malaise trap, ST: Sticky trap, Mx: Mexico. Do: Dominic Republic.

| Dolichopodidae | PT | EO | E | M | ST | Mx Amber | Do amber |
| --- | --- | --- | --- | --- | --- | --- | --- |
| *Achalchus* | 0 | 0 | 0 | 1 | 0 | 0 | 2 |
| *Achradocera* | 0 | 0 | 0 | 0 | 7 | 0 | 0 |
| *Amblypsilopus* | 0 | 0 | 0 | 6 | 12 | 6 | 1 |
| *Asyndetus* | 0 | 0 | 0 | 0 | 46 | 0 | 1 |
| *Corindia* | 0 | 0 | 0 | 0 | 1 | 0 | 0 |
| *Chrysotus* | 0 | 1 | 3 | 54 | 232 | 0 | 5 |
| *Condylostylus* | 0 | 0 | 0 | 4 | 0 | 0 | 4 |
| *Diaphorus* | 1 | 0 | 0 | 6 | 20 | 0 | 6 |
| *Dominicomyia* | 0 | 0 | 0 | 10 | 0 | 0 | 10 |
| *Mesorhaga* | 0 | 0 | 0 | 1 | 0 | 2 | 2 |
| *Micromorphus* | 0 | 0 | 0 | 4 | 2 | 0 | 0 |
| *Neurigona* | 0 | 0 | 0 | 1 | 1 | 0 | 0 |
| *Medetera* | 0 | 0 | 1 | 2 | 27 | 2 | 19 |
| *Paraclius* | 0 | 0 | 0 | 0 | 2 | 0 | 0 |
| *Peloropeodes* | 0 | 1 | 0 | 2 | 3 | 2 | 0 |
| *Sciapus* | 0 | 0 | 0 | 2 | 0 | 0 | 2 |
| *Thrypticus* | 0 | 0 | 0 | 8 | 0 | 0 | 0 |
| *Viridigona* | 0 | 0 | 0 | 5 | 0 | 0 | 0 |
| new genus & species | 0 | 0 | 0 | 0 | 0 | 6 | 3 |
| Unidentified | 0 | 0 | 1 | 0 | 3 | 5 | 0 |
